# Variations in activin receptor, inhibin/activin subunit and follistatin mRNAs in human prostate tumour tissues

**DOI:** 10.1054/bjoc.1999.0886

**Published:** 1999-12-08

**Authors:** R H N van Schaik, C D J Wierikx, M A Timmerman, M H Oomen, W M van Weerden, T H van der Kwast, G J van Steenbrugge, F H de Jong

**Affiliations:** Departments of Endocrinology & Reproduction, Urology, Pathology, Erasmus University Rotterdam, Rotterdam, The Netherlands

**Keywords:** activin receptor, activin, inhibin, follistatin, prostate cancer

## Abstract

The possible role of activin in the regulation of malignant prostatic growth was studied using RNAase protection assays of activin receptors, inhibin/activin subunits and follistatin mRNAs in the human prostatic carcinoma cell lines LNCaP-FGC, -R and -LNO, in human prostatic carcinoma xenografts and in human prostatic tissue. Activin receptor types IA (ActRIA), IB (ActRIB), IIA (ActRIIA) and IIB (ActRIIB) mRNAs were generally expressed in prostate pithelial cells, with significantly lower levels of ActRIB mRNA in prostate tumour aterial when compared to non-malignant tissue (*P*< 0.05; Mann–Whitney *U* -test). Inhibin/activin βA- and βB-subunit mRNA expression was also found in prostate tissue. Androgen-independent xenografts expressed significantly lower amounts of βB-subunit mRNA when compared to androgen-dependent xenografts (*P*< 0.05). While βB-subunit mRNA was expressed by LNCaP-FGC and -LNO cells, virtually no expression was found in the androgen-independent LNCaP-R line. Inhibin α-subunit mRNA levels were low or undetectable in all samples investigated. Follistatin mRNA was undetectable in LNCaP-sublines, while low levels were found in prostatic tissues. In androgen-independent LNCaP-R cells, activin inhibited cell growth in a dose-dependent manner. These results suggest that prostate tumour progression is accompanied by a decrease of the inhibitory effect of locally produced activin by either a decrease in the expression of activin βB-subunit mRNA or by a decrease of ActRIB mRNA levels. © 2000 Cancer Research Campaign


*British Journal of Cancer* (2000) **82**(1), 112–117
© 2000 Cancer Research Campaign
Article no. bjoc.1999.0886

### Variations in activin receptor, inhibin/activin subunit and follistatin mRNAs in human prostate tumour tissues

**RHN van Schaik[1], CDJ Wierikx[1], MA Timmerman[1], MH Oomen[2], WM van Weerden[2], TH van der Kwast[3], GJ van Steenbrugge[2] and FH de Jong[1]**

Departments of [1]Endocrinology & Reproduction, [2]Urology and [3]Pathology, Erasmus University Rotterdam, Rotterdam, The Netherlands

**Summary** The possible role of activin in the regulation of malignant prostatic growth was studied using RNAase protection assays of activin receptors, inhibin/activin subunits and follistatin mRNAs in the human prostatic carcinoma cell lines LNCaP-FGC, -R and -LNO, in human prostatic carcinoma xenografts and in human prostatic tissue. Activin receptor types IA (ActRIA), IB (ActRIB), IIA (ActRIIA) and IIB (ActRIIB) mRNAs were generally expressed in prostate epithelial cells, with significantly lower levels of ActRIB mRNA in prostate tumour material when compared to non-malignant tissue ($P < 0.05$; Mann–Whitney *U*-test). Inhibin/activin βA- and βB-subunit mRNA expression was also found in prostate tissue. Androgen-independent xenografts expressed significantly lower amounts of βB-subunit mRNA when compared to androgen-dependent xenografts ($P < 0.05$). While βB-subunit mRNA was expressed by LNCaP-FGC and -LNO cells, virtually no expression was found in the androgen-independent LNCaP-R line. Inhibin α-subunit mRNA levels were low or undetectable in all samples investigated. Follistatin mRNA was undetectable in LNCaP-sublines, while low levels were found in prostatic tissues. In androgen-independent LNCaP-R cells, activin inhibited cell growth in a dose-dependent manner. These results suggest that prostate tumour progression is accompanied by a decrease of the inhibitory effect of locally produced activin by either a decrease in the expression of activin βB-subunit mRNA or by a decrease of ActRIB mRNA levels. © 2000 Cancer Research Campaign

**Keywords:** activin receptor; activin; inhibin; follistatin; prostate cancer

Clinical prostatic cancer develops in approximately one in every 11 males in the Western world. It has become the most commonly diagnosed cancer in men, and is presently the second leading cause of cancer deaths. Localized prostate cancer can be cured by radical prostatectomy, whereas patients suffering from extensive or metastasized tumours usually receive androgen ablation therapy. In spite of a high initial response rate of patients to this endocrine treatment, its benefit is limited by tumour cells becoming androgen-independent for their growth, leading to uncontrolled proliferation. Peptide growth factors can also influence prostatic growth (Ilio et al, 1995). Activins, which are composed of two β-subunits (βA and/or βB) and their antagonists, the inhibins (combinations of an α- and either of the two β-subunits) are members of the transforming growth factor (TGF)-β superfamily of peptide growth- and differentiation factors. They influence proliferation in a variety of cell systems (Gonzalez-Manchon and Vale, 1989; Hedger et al, 1989; Hashimoto et al, 1990). Activin exerts its effect by binding to one of two activin type II receptors (ActRIIA or ActRIIB), which are membrane-spanning serine/threonine kinase receptors (Mathews, 1994). Combination of the activin–ActRII complex with either one of the two activin receptors type I (ActRIA or ActRIB) initiates a signalling cascade (ten Dijke et al, 1994), which involves specific transcription factors, the Smads (Massagué, 1996). The detection of ActRIIA and ActRIIB receptor mRNA expression in the rat prostate (Feng et al, 1993) suggests an involvement of activin in the regulation of prostatic growth. In addition, the human prostatic cancer cell lines LNCaP and DU-145 express at least one type of activin type I receptors, as demonstrated by reverse transcription polymerase chain reaction (RT-PCR) (Batres et al, 1995; Furst et al, 1995; Ying et al, 1995). Recently, the presence of immuno-reactive inhibin/activin subunits was demonstrated in the rat prostate (Risbridger et al, 1996) and in prostatic tissue of men with benign prostatic hyperplasia (Thomas et al, 1998). Finally, activin has an inhibitory effect on the growth of the human prostatic carcinoma cancer cell line LNCaP (Dalkin et al, 1996; Wang et al, 1996*b*; McPherson et al, 1997; Zhang et al, 1997*a*, 1997*b*), indicating that the activin/inhibin system can play a role in the control of prostate epithelial cell proliferation. Changes in expression of inhibin/activin subunits, activin receptors or the activin-binding protein follistatin may therefore influence growth of prostate epithelial cells. On the basis of these data, we hypothesized that transcript levels for activin β-subunits or activin receptors might be lower in tumour material when compared to normal prostatic tissue (in the case of clinical material) and in androgen-independent cell lines and xenografts when compared to their androgen-dependent counterparts.

Therefore we measured the mRNA expression levels of activin type I and type II receptors, of inhibin α- and inhibin/activin β-subunits and of follistatin by RNAase protection analyses in androgen-dependent and androgen-independent human prostatic xenografts, in various sublines of the human prostatic cell line LNCaP, and in non-malignant and malignant prostate tissues.

*Received 13 October 1998*
*Revised 17 May 1999*
*Accepted 25 May 1999*

*Correspondence to:* RHN van Schaik, Department of Clinical Chemistry, University Hospital Rotterdam – Dijkzigt, PO Box 2040, 3000 CA Rotterdam, The Netherlands

### MATERIALS AND METHODS

### Cell lines, xenografts and primary tumours

The androgen-dependent LNCaP-FGC (FGC) and the androgen-independent LNCaP-r (R) and LNCaP-LNO (LNO) sublines have

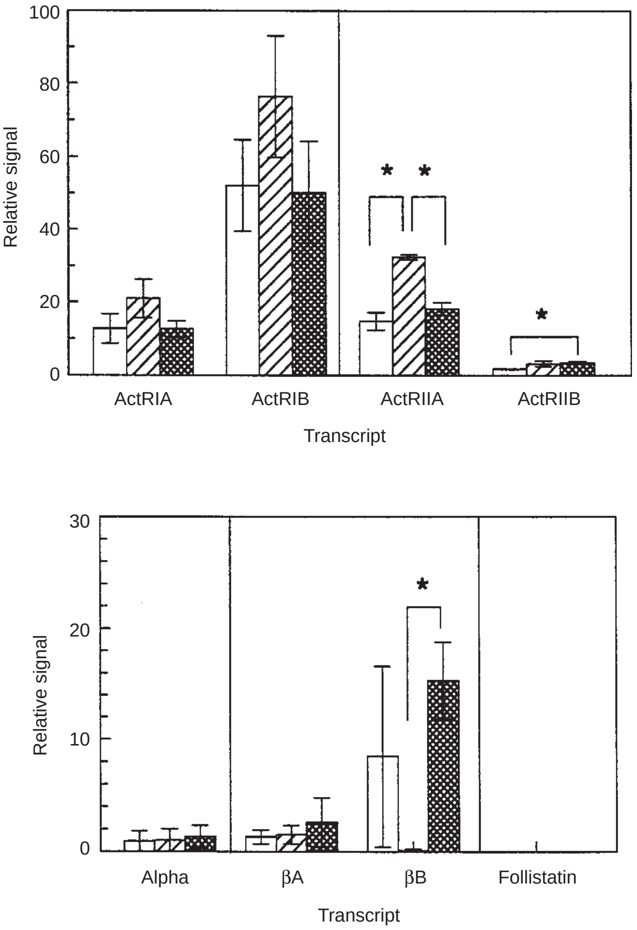

**Figure 1** Expression of activin receptor, inhibin subunit and follistatin mRNAs in androgen-dependent LNCaP-FGC (open bars), and androgen-independent LNCaP-R (hatched bars) and LNCaP-LNO cells (cross-hatched bars). Signals are expressed in arbitrary units, relative to actin. Mean values ± s.e.m. are shown for three independent experiments. Significant differences ($P < 0.05$) have been indicated with asterisks

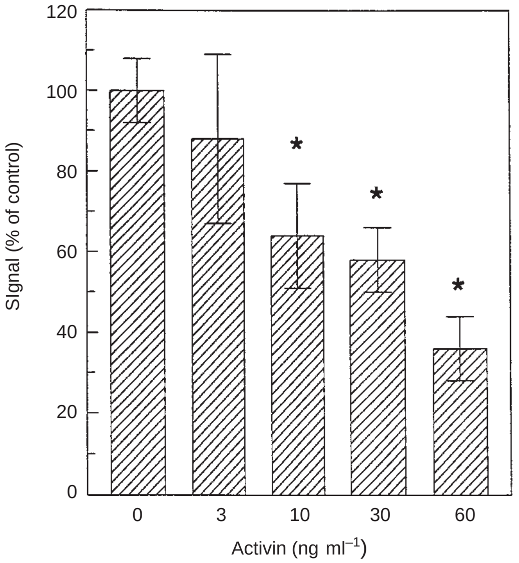

**Figure 2** Effect of activin on the proliferation of the androgen-independent cell line LNCaP-R. Cells were grown for 5 days in DCC-treated medium with the indicated amounts of activin ($n = 8$). Growth was determined in a colourimetic (MTT) assay, and plotted relative to cells grown in DCC, which was set at 100%. Significant differences ($P < 0.05$) in proliferation from control (DCC without activin) are marked with an asterisk been derived from the original LNCaP cell line (Horoszewicz et al, 1983). FGC and LNO (van Steenbrugge et al, 1991) were kindly provided by Dr JS Horoszewicz, subline FGC being identical to the LNCaP line available through the American Type Culture Collection (ATCC). The R-subline (Hasenson et al, 1985) was a gift from Dr M Hasenson (Karolinska Institute, Sweden). FGC and R were routinely cultured in RPMI medium, supplemented with 10% fetal calf serum (FCS), glutamine and antibiotics. For culturing LNO, regular FCS was replaced by 5% dextran-coated charcoal (DCC)-treated, i.e. androgen-depleted, FCS. In order to study effects of activin on cell proliferation, R cells were grown for 5 days in DCC-treated medium with the indicated amounts of activin A (Innogenetics, Ghent, Belgium). Growth was determined with a colourimetric (MTT) assay, as described earlier (Romijn et al, 1988).

The prostate tumour xenografts used in this study are the androgen-dependent PC-82 (Hoehn et al, 1980), PC-295, PC-310, PC-329 (van Weerden et al, 1996) and the androgen-independent PC-133, PC-135 (van Steenbrugge, unpublished results), PC-324, PC 339 and PC-374 (van Weerden et al, 1996).

Primary tumour material was obtained after radical prostatectomy in 24 patients. From 20 sections of paraffin-embedded tissue to be used for RNA isolation, sections 1, 10 and 20 were used for histological examination. Based upon these observations, 12 samples were classified as normal (i.e. non-malignant) and 12 as carcinoma (Gleason scores: G-6 (1), G-7 (1), G-8 (5), G-9 (2), G-10 (3)).

### RNA isolation and RNAase protection assays

Frozen tissue (–80°C) was pulverized in liquid nitrogen, followed by RNA isolation using TRIzol reagent (Gibco-BRL, Gaithersburg, MD, USA), according to the manufactures protocol. For the cell lines, cells were washed once in phospate-buffered saline (PBS) after which TRIzol reagent was added to the culture flasks. RNA was dissolved in RNAase-free water and its concentration and purity were determined by optical density measurement at 260 and 280 nm.

cDNA clones for the specific human transcripts (van Schaik et al, 1997) were digested with appropriate restriction enzymes and $^{32}$P-labelled RNA probes were made by transcription in the presence of $^{32}$P-UTP, using T3 or T7 RNA polymerase (Stratagene, La Jolla, CA, USA). The cRNA probes protect nucleotides 126–411 for the inhibin α-subunit (Mayo et al, 1986), nucleotides 702–946 for the βA-subunit (Mason et al, 1986), nucleotides 845–1073 for the βB-subunit (Mason et al,1986) and nucleotides 508–819 for the follistatin transcript. The latter probe therefore detects mRNAs, which can be translated into both follistatin-288 and follistatin-315. For the activin receptors, the cRNA probes protect nucleotides 1482–1692 for ActRIA (ten Dijke et al, 1993), nucleotides 191–361 for ActRIB (GenBank, accession number Z22536), nucleotides 132–791 for ActRIIA (Donaldson et al, 1992) and nucleotides 429–947 for ActRIIB (Hildén et al, 1994). For human γ-actin, a probe corresponding to nucleotides 1207–1337 (Erba et al, 1986) was used. RNAase protection assays were performed as described by Sambrook et al (1989). RNAase treatment was performed using

*© 2000 Cancer Research Campaign* 

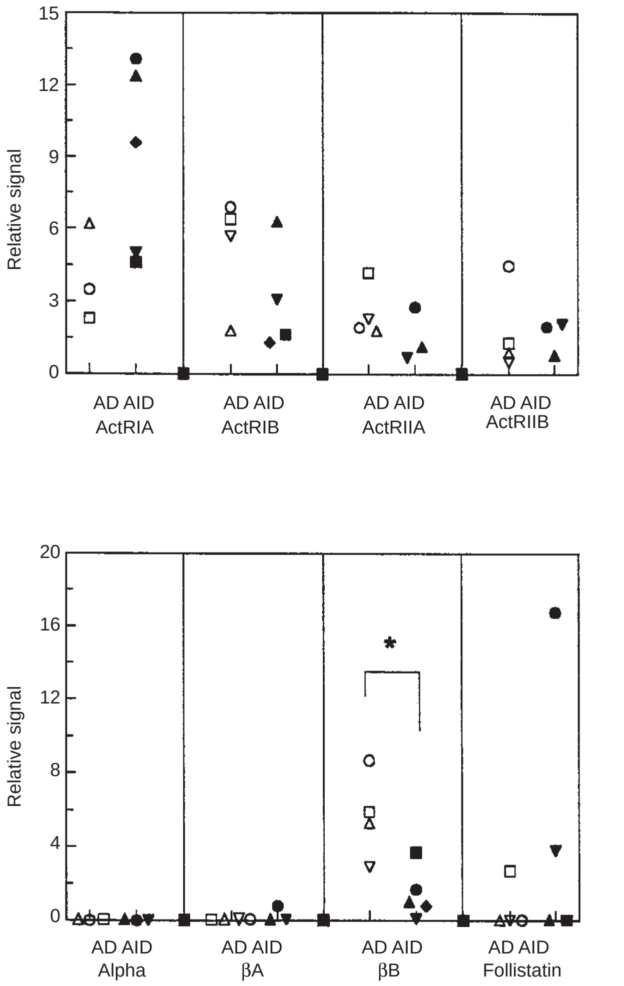

**Figure 3** Relative expression of mRNA encoding activin receptors, inhibin subunits and follistatin in human androgen-dependent (AD): PC-82 (▽), PC-295 (△), PC-310 (○), PC-329 (□); and androgen-independent (AID) xenografts: PC-133 (■), PC-135 (◆), PC324 (●), PC-339 (▼), PC-374 (▲), as measured by RNAase protection. Signals are expressed in arbitrary units, relative to actin. Significant differences ($P < 0.05$) are indicated with an asterisk

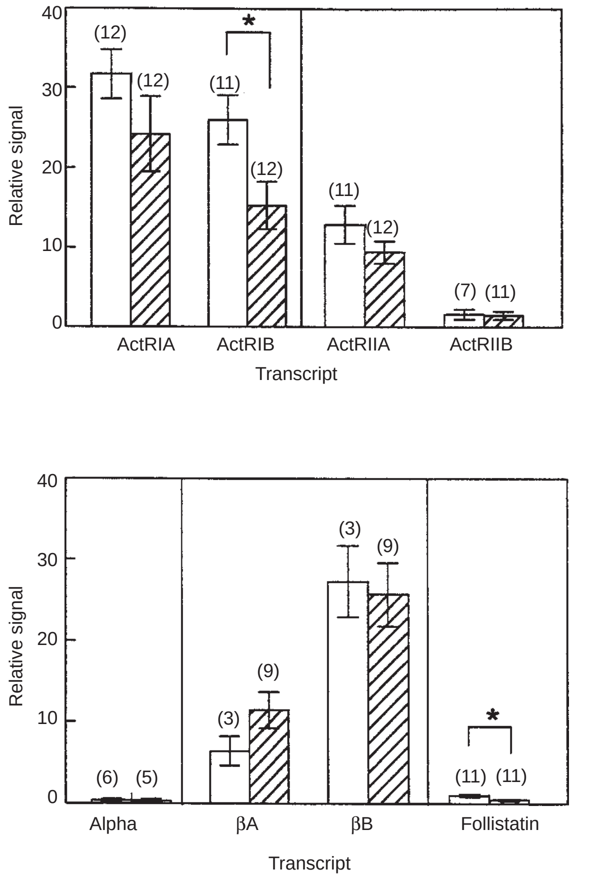

**Figure 4** Relative expression of mRNA encoding activin receptors, inhibin subunits and follistatin in non-malignant (open bars) and malignant (hatched bars) clinical prostatic tissue, as measured by RNAase protection. Signals are expressed in arbitrary units, relative to actin. Significant differences ($P < 0.05$) between the non-malignant and malignant clinical samples are indicated with an asterisk. The numbers in parentheses indicate the number of samples determined in each group

100 U $ml^{-1}$ RNAase T1 (Boehringer, Mannheim, Germany) in combination with 10 µg $ml^{-1}$ RNAase A (Boehringer). Hybridization temperature was 42°C. Routinely, 5 µg RNA was analysed. In each run, positive and negative controls were included. Results were quantified using a PhosphorImager (Molecular Dynamics/B&L Systems, Maarssen, The Netherlands) and data are expressed relative to γ-actin. Unless stated otherwise, mean values ± s.e.m. are given and comparisons between groups were made using the Mann–Whitney *U*-test. *P*-values < 0.05 were interpreted as indicating a significant difference.

### RESULTS

### LNCaP sublines

In three independent experiments, we found that the androgen-dependent FGC- and the androgen-independent R- and LNO-cell lines expressed activin type I (ActRIA and ActRIB) and type II (ActRIIA and ActRIIB) receptor mRNAs at comparable levels (Figure 1). For ActRIIA mRNA expression, significantly higher levels were found in the R-line when compared to the FGC- and LNO-lines. Furthermore, a small but significant difference was observed between the FGC- and LNO-line for the ActRIIB mRNA levels. The FGC and LNO cell lines express inhibin/activin βB-subunit mRNA, while in the R-line βB-subunit mRNA was virtually absent. We found low inhibin/activin βA-subunit mRNA levels in all three sublines, while expression of inhibin α-subunit mRNA was at the detection limit of our assay. Follistatin mRNA expression was not detectable.

The functionality of the activin signalling pathway in the LNCaP sublines was investigated by growing the androgen-independent R cells in the presence of varying amounts of activin. The cells were significantly inhibited in their proliferation when incubated on DCC-medium in the presence of activin ($P < 0.05$) (Figure 2).

### Prostatic carcinoma xenografts

In tissue specimens of well defined androgen-dependent and androgen-independent xenograft models, we quantitatively investigated expression of inhibin/activin subunits, activin receptors and follistatin mRNAs. All xenografts studied expressed activin type I (ActRIA and ActRIB) and type II (ActRIIA and ActRIIB) receptors (Figure 3). Inhibin/activin βA-subunit expression was not detectable in the androgen-dependent xenografts, while of the androgen-independent xenografts only PC-324 showed a low level of expression. Inhibin/activin βB-subunit mRNA, however, was abundantly expressed in the androgen-dependent xenografts. This expression level was found to be significantly lower ($P < 0.05$) in the androgen-independent tumours. Follistatin mRNA was expressed with a large variation, ranging from undetectable to a relatively high (16.8 relative units (RU)) expression in the androgen-independent xenograft PC-324. We could not detect inhibin α-subunit mRNA expression in the xenograft samples.

### Clinical prostatic carcinomas

In all clinical prostate samples investigated, expression of activin type I (ActRIA and ActRIB) and type II (ActRIIA) receptors was found, while ActRIIB expression was detected in four of seven non-malignant prostatic tissues and in nine of eleven samples of prostate carcinomas. The carcinomas expressed significantly lower levels of ActRIB mRNA when compared to the non-malignant tissues (15.3 ± 3.0 vs 26.0 ± 3.1 RU respectively; $P < 0.05$) (Figure 4). Inhibin/activin βA- and βB-subunit mRNAs were expressed at comparable levels in non-malignant and carcinomatous samples. Inhibin α-subunit mRNA expression was low or not detectable. Follistatin mRNA expression was generally found at low expression levels, and was significantly lower in the carcinomas when compared to non-malignant tissue ($P < 0.05$).

### DISCUSSION

### Activin receptors

We measured mRNA expression levels of activin receptors type IA, IB and type IIA, IIB in human prostate cancer cells, either in culture, in xenografts or clinical tissues. In the three sublines of LNCaP, significant differences were observed in the expression of ActRIIA and ActRIIB mRNA. In prostatic carcinoma xenografts we demonstrated expression of all known activin type I and activin type II receptors. No significant differences in expression of activin receptors were observed when androgen-dependent and androgen-independent xenografts were compared. In tissue obtained after radical prostatectomy, in which the neoplastic grade of the samples was determined microscopically to range from non-malignant to Gleason grade 10, expression of ActRIA and ActRIB mRNA was found. Transcript levels for ActRIB were significantly lower in the carcinomas when compared to the non-malignant tissues. This implies a decreased sensitivity to growth inhibition by activin: it was shown that ActRIB is responsible for inhibition of proliferation by activin, rather than ActRIA (Cárcamo et al, 1994). No correlation was observed between Gleason grade and ActRIB mRNA levels within this limited series. Expression of ActRIIA mRNA was demonstrated in all samples, but ActRIIB mRNA transcripts were undetectable in a number of them. These results corroborate and significantly extend previous reports using the reverse transcriptase polymerase chain reaction (RT-PCR) for the qualitative detection of expression of activin receptors in human prostate cancer cell lines (Batres et al, 1995; Furst et al, 1995; Dalkin et al, 1996), and are in line with the detection of ActRIIA and IIB transcripts in the rat prostate (Feng et al, 1993) and recently of ActRIIA mRNA in benign prostate hyperplasia (Thomas et al, 1998). Remarkably, the relative signal for ActRIA was lower than that for ActRIB in the LNCaP cell lines while in most of the xenografts relatively more ActRIA was expressed than ActRIB. In the clinical prostate samples expression of ActRIA and of ActRIB were comparable; this may have been caused by the presence of stromal tissue in these samples.

The combined expression of type I and type II receptors in human prostatic epithelial cells indicates that a functional activin receptor complex can be formed in the presence of activin, thereby enabling activin to have an effect on these cells. We confirmed this in androgen-independent R cells. These results are in line with data reported on androgen-dependent LNCaP cells (Dalkin et al, 1996; Wang et al, 1996*b*), but differ with results obtained with the androgen-independent cell lines PC3 and DU-145 (Ying et al, 1997). Possibly, the defect in the retinoblastoma gene product in DU-145 (Rubin et al, 1991) and the expression of relatively large amounts of follistatin by PC3 cells (Wang et al, 1996*a*) or the presence of a mutated p53 gene in PC3 cells (Rubin et al, 1991) are responsible for this lack of response to activin treatment.

### Activin/inhibin subunits and follistatin

In addition to activin receptor expression, we quantified mRNA levels for inhibin/activin β-subunits in prostate tumour cell lines, in prostate tumour xenografts and in samples of non-malignant and malignant prostatic tissues. Expression of predominantly βB-subunit mRNA was found in the FGC cell line, but this expression was virtually absent in the androgen-independent R-line. Decreased production of activin might lead to a growth advantage; the R-line is indeed still sensitive to growth inhibition by activin (Figure 2). However, the other androgen-independent cell line in our study (LNO) did not show this decreased βB-subunit expression, indicating that not all androgen-independent cell lines have low levels of activin. For the FGC-subline, we found variable results for inhibin/activin βB-subunit expression in three independent experiments, resulting in a relative large variation, which cannot be explained at this moment. The three independent experiments were performed with relatively large time intervals; therefore we hypothesize that, at least for this subline, variable conditions of e.g. cell density or passage number, may have caused the observed variation. Qualitative expression of βB-subunit mRNA in LNCaP has been reported earlier, using RT-PCR (Batres et al, 1995; Furst et al, 1995). Dalkin et al (1996) investigated the effect of endogenous activin on proliferation of LNCaP-FGC cells by addition of follistatin and found an increase in proliferation after 24 h treatment confirming endogenous activin production. Low, but detectable inhibin α-subunit mRNA levels were found in the RNAase protection assay for the LNCaP sublines, whereas the absence of inhibin α-subunit mRNA in LNCaP cells using RT-PCR has been reported earlier (Batres et al, 1995). It is unclear whether this difference is caused by subtle differences in properties of the LNCaP cell lines used, or culture conditions applied. On the other hand, the RT-PCR assay may not have functioned optimally, which is difficult to assess due to the absence of a positive control for the inhibin α-subunit mRNA RT-PCR (Batres et al, 1995). Inhibin immunoreactivity was found to be absent in LNCaP cells (Batres et al, 1995), suggesting that inhibin production in FGC cells is indeed very low. In the xenografts, βA-subunit expression was not detectable in the androgen-dependent samples and was only detected at low levels in one out of three androgen-independent tumours. RNAase protection will only measure

© 2000 Cancer Research Campaign expression in the epithelial component of the xenografts since stromal cells express the murine genes which, with the exception of ActRIIA mRNA, are not protected by the human cRNA probes (RHN van Schaik, unpublished results). This suggests that the stromal cells may be responsible for the inhibin βA-subunit mRNA expression in the prostate. The androgen-independent xenografts had significantly lower expression levels of inhibin/activin βB-subunit mRNA when compared to the androgen-dependent samples, indicating a decrease in activin production with increasing malignancy. In the clinical samples we found a relatively high expression of both βA- and βB-subunit transcripts. Inhibin α-subunit mRNA levels in non-malignant and malignant prostate tissues were low or undetectable as determined by RNAase protection. Using in situ hybridization and immunohistochemistry on BPH- and prostate cancer samples, expression of inhibin α-subunit mRNA was found in prostate epithelial and basal cells, which was absent in malignant regions of tissue from men with high grade prostate cancer (Mellor et al, 1998). We did not find a significant difference in the expression of inhibin α-subunit transcript levels when non-malignant ($0.3 \pm 0.3$ RU) and tumour samples ($0.4 \pm 0.2$ RU) were compared, although this may be due to the very low expression levels detected in our study. We found a significantly higher expression of follistatin mRNA in the non-malignant samples when compared to the carcinomatous tissues. Because follistatin mRNA expression was undetectable by RNAase protection in the LNCaP sublines, follistatin might be expressed in stromal cells rather than in the epithelial cells. In fact, follistatin immunoreactivity was recently reported to be localized in fibroblastic stroma (Thomas et al, 1998). Human follistatin mRNA expression was also found in three out of nine xenografts studied, indicating that the epithelial cells are also capable of expressing follistatin mRNA. In addition, the human prostatic carcinoma cell line PC3 expresses large amounts of follistatin (Wang et al, 1996*a*) while also follistatin mRNA could be detected in LNCaP cells using RT-PCR (Dalkin et al, 1996). All in all, these data indicate that prostate epithelial cells can express follistatin, and suggest that the expression level in LNCaP cells is relatively low.

Our results on mRNA expression indicate that autocrine growth control by activin can occur in the prostate, and that malignant cells may escape growth inhibition by activin by a decrease in mRNA expression levels for the activin βB-subunit or for the activin receptor type IB. Decreased activin signalling may lead to increased cell proliferation and inhibition of apoptosis of prostate epithelial cells (Zhang et al, 1997*a*), thereby facilitating prostate tumour development. Future experiments need to establish to what extent the differences found in mRNA levels can be translated to the protein level and can lead to differences in the behaviour of the tumours.

### ACKNOWLEDGEMENTS

Innogenetics, Ghent, Belgium, is greatly acknowledged for the supply of activin A. This work was supported by a grant from the Dutch Cancer Society (IKMN 92-75).

### REFERENCES

Batres Y, Zhang Z and Ying SY (1995) Expression of activins and activin receptor messenger RNAs in LNCaP cells, a human prostatic adenocarcinoma cell line. *Int J Oncol* **6**: 1185–1188

Cárcamo J, Weis FMB, Ventura F, Wieser R, Wrana JL, Attisano L and Massagué J (1994) Type I receptors specify growth-inhibitory and transcriptional responses to transforming growth factor β and activin. *Mol Cell Biol* **14**: 3810–3821

Dalkin AC, Gilrain JT, Bradshaw D and Myers CE (1996) Activin inhibition of prostate cancer cell growth: selective actions on androgen-responsive LNCaP cells. *Endocrinology* **137**: 5230–5235

Donaldson CJ, Mathews LS and Vale WW (1992) Molecular cloning and binding properties of the human type II activin receptor. *Biochem Biophys Res Commun* **184**: 310–316

Erba HP, Gunning P and Kedes L (1986) Nucleotide sequence of the human γ cytoskeletal actin mRNA: anomalous evolution of vertebrate non-muscle actin genes. *Nucleic Acids Res* **14**: 5275–5294

Feng ZM, Madigan MB and Chen CLC (1993) Expression of type II activin receptor genes in the male and female reproductive tissues of the rat. *Endocrinology* **132**: 2593–2600

Furst BA, Zhang Z and Ying SY (1995) Expression of activin and activin receptors in human prostatic carcinoma cell line DU145. *Int J Oncol* **7**: 239–243

Gonzalez-Manchon C and Vale W (1989) Activin-A, inhibin and transforming growth factor-β modulate growth of two gonadal cell lines. *Endocrinology* **125**: 1666–1672

Hasenson M, Hartley-Asp B, Kihlfors C, Lundin A, Gustafsson JA and Pousette A (1985) Effect of hormones on growth and ATP content of a human prostatic carcinoma cell line, LNCaP-r. *Prostate* **7**: 183–194

Hashimoto M, Kondo S, Sakurai T, Etoh Y, Shibai H and Muramatsu M (1990) Activin/EDF as an inhibitor of neural differentiation. *Biochem Biophys Res Commun* **173**: 193–200

Hedger MP, Drummond AE, Robertson DM, Risbridger GP and de Kretser DM (1989) Inhibin and activin regulate [$^3$H]thymidine uptake by rat thymocytes and 3T3 cells in vitro. *Mol Cell Endocrinol* **61**: 133–138

Hildén K, Tuuri T, Erämaa M and Ritvos O (1994) Expression of type II activin receptor genes during differentiation of human K562 cells and cDNA cloning of the human type IIB activin receptor. *Blood* **83**: 2163–2170

Hoehn W, Schröder FH, Riemann JF, Joebsis AC and Hermanek P (1980) Human prostatic adenocarcinoma: some characteristics of a serially transplantable line in nude mice (PC 82). *Prostate* **1**: 95–104

Horoszewicz JS, Leong SS, Kawinski E, Karr JP, Rosenthal H, Chu TM, Mirand EA and Murphy GP (1983) LNCaP model of human prostatic carcinoma. *Cancer Res* **43**: 1809–1818

Ilio KY, Sensibar JA and Lee C (1995) Effect of TGF-β1, TGF-α, and EGF on cell proliferation and cell death in rat ventral prostatic epithelial cells in culture. *J Androl* **16**: 482–490

McPherson SJ, Thomas TZ, Wang H, Gurusinghe CJ and Risbridger GP (1997) Growth inhibitory response to activin A and B by human prostate tumour cell lines, LNCaP and DU145. *J Endocrinol* **154**: 535–545

Mason AJ, Biall HD and Seeburg PH (1986) Structure of two human ovarian inhibins. *Biochem Biophys Res Commun* **135**: 957–964

Massagué J (1996) TGFβ signaling: receptors, transducers, and Mad proteins. *Cell* **85**: 947–950

Mathews LS (1994) Activin receptors and cellular signaling by the receptor serine kinase family. *Endocr Rev* **15**: 310–325

Mayo KE, Cerelli GM, Spiess J, Rivier J, Rosenfeld MG, Evans RM and Vale W (1986) Inhibin A-subunit cDNAs from porcine ovary and human placenta. *Proc Natl Acad Sci USA* **83**: 5849–5853

Mellor SL, Richards MG, Pedersen JS, Robertson DM and Risbridger GP (1998) Loss of expression and localization of inhibin α-subunit in high grade prostate cancer. *J Clin Endocrin Metab* **83**: 969–975

Risbridger GP, Thomas T, Gurusinghe CJ and McFarlane JR (1996) Inhibin-related proteins in rat prostate. *J Endocrinol* **149**: 93–99

Romijn JC, Verkoelen, CF and Schröder FH (1988) Application of the MTT assay to human prostate cancer cell lines in vitro: establishment of test conditions and assessment of hormone-stimulated growth and drug-induced cytostatic and cytotoxic effects. *Prostate* **12**: 99–110

Rubin SJ, Hallahan DE, Ashman CR, Brachman DG, Beckett MA, Virudachalam S, Yandell DW and Weichselbaum RR (1991) Two prostate carcinoma cell lines demonstrate abnormalities in tumor suppressor genes. *J Surg Oncol* **46**: 31–36

Sambrook J, Fritsch EF and Maniatis T (1989) *Molecular Cloning: a Laboratory Manual*. Cold Spring Harbor Press: New York ten Dijke P, Ichijo H, Franzén P, Schulz P, Saras J, Toyoshima H, Heldin CH and Miyazono K (1993) Activin receptor-like kinases: a novel subclass of cell-surface receptors with predicted serine/threonine kinase activity. *Oncogene* **8**: 2879–2887

ten Dijke P, Franzen P, Yamashita H, Ichijo H, Heldin CH and Miyazono K (1994) Serine/threonine kinase receptors. *Prog Growth Factor Res* **5**: 55–72

 *© 2000 Cancer Research Campaign*

Thomas TZ, Chapman SM, Hong W, Gurusinghe C, Mellor SL, Fletcher R, Pedersen J and Risbridger GP (1998) Inhibins, activins, and follistatins: expression of mRNAs and cellular localization in tissues from men with benign prostatic hyperplasia. *Prostate* **34**: 34–43

van Schaik RHN, Wierikx CDJ, Looijenga LHJ, Oosterhuis JW and de Jong FH (1997) Human testicular germ cell tumours express inhibin subunits, activin receptors and follistatin mRNAs. *Br J Cancer* **76**: 1191–1198

van Steenbrugge GJ, van Uffelen CJ, Bolt J and Schröder FH (1991) The human prostatic cancer cell line LNCaP and its derived sublines: an in vitro model for the study of androgen sensitivity. *J Steroid Biochem Mol Biol* **40**: 207–214

van Weerden WM, de Ridder CM, Verdaasdonk CL, Romijn JC, van der Kwast TH, Schröder FH and van Steenbrugge GJ (1996) Development of seven new human prostate tumor xenograft models and their histopathological characterization. *Am J Pathol* **149**: 1055–1062

Wang QF, Schneyer AL, Crowley WF and Sluss PM (1996*a*) Overexpression of follistatin by a human prostate cancer cell line, PC-3. In: *10th International Congress of Endocrinology*, pp. 8823 (Abstract P3-276): San Francisco CA, USA.

Wang QF, Tilly KI, Tilly JL, Preffer F, Schneyer AL, Crowley WF, Jr. and Sluss PM (1996*b*) Activin inhibits basal and androgen-stimulated proliferation and induces apoptosis in the human prostatic cancer cell line, LNCaP. *Endocrinology* **137**: 5476–5483

Ying SY, Zhang Z and Xing W (1995) Expression of activin and activin receptors in PC3 human prostatic cancer cells. *Int J Oncol* **6**: 601–606

Ying SY, Zhang Z, Batres Y, Zhao Y, Lin SL and Li G (1997) p53 is involved in the inhibition of cell proliferation mediated by activin A in cultured human prostate cancer LNCaP cells. *Int J Oncol* **11**: 591–595

Zhang Z, Zheng J, Zhao Y, Li G, Batres Y, Luo MP, Wan M, Ying SY (1997*a*). Overexpression of activin A inhibits growth, induces apoptosis, and suppresses tumorigenicity in an androgen-sensitive human prostate cancer cell line LNCaP. *Int J Oncol* **11**: 727–736

Zhang Z, Zhao Y, Batres Y, Lin MF and Ying SY (1997*b*) Regulation of growth and prostatic marker expression by activin A in an androgen-sensitive prostate cancer cell line LNCAP. *Biochem Biophys Res Commun* **234**: 362–365

*© 2000 Cancer Research Campaign*
